# Funis Presentation

**Published:** 1860-05

**Authors:** S. Brandies

**Affiliations:** Louisville, Ky.


### FUNIS PRESENTATION.

### THREE CASES TREATED SUCCESSFULLY BY THE POSTURE METHOD.

BY S. BRANDIES, M. D., LOUISVILLE, KY.

Case I.—Mrs. Katharine Rehm, aged thirty, a native of Germany, and who has borne four children, (one of which was still-born in conseqenc of prolapse of the funis,) was seized with her fifth labor on the first day of July, 1858, at 4 o'clock, A. M. At 5 o'clock the membranes ruptured, followed by a full gush of liquor amnii, which carried forward with it a long loop of the funis and the right hand. The midwife in attendance, having discovered the mischief, sent immediately for my assistance.

I reached the patient within ten minutes, and found the funis, feebly pulsating, outside of the genitals, the hand within, and the head balloting high above the entrance of the pelvis. Remembering a plan suggested by Dr. Thomas, of New York, I forthwith placed the parturient on her knees and elbows, supporting the body with pillows in such a way that the pelvis was a good deal higher than the chest.

With slow and careful manipulations I succeeded in replacing both funis and arm far beyond the head, while I kept my hand within the cavity of the womb in order to prevent a further prolapse. Strong labor pains set in, and, soon after, the head engaged so firmly in the superior entrance of the pelvis that all apprehension of a procedentia of the funis vanished. The patient was now placed on her back, labor proceeded rapidly, and three-quarters of an hour later a living child (a boy) was born.

The patient did very well afterwards. The placenta was spontaneously expelled, there was very little after-pain, and recovery took place rapidly. The child is now eighteen months old, vigorous and healthy.

Case II.—Mrs. Katharine Rapp, aged thirty-six, a native of Germany, a stout and healthy woman, and the mother of four children, was taken with labor pains at eight o'clock, P. M., October 12th, 1859. Soon after the arrival of the midwife the membranes ruptured, and a loop of the funis and the hand presented. I was summoned to the case at 11 o'clock, P. M. On exomination, I found the midwife's diagnosis correct: the hand was the left one; the loop of the funis, still pulsating, was about three inches long; whilst the head was high above the entrance of the pelvis. The method described in Case I, was immediately carried into operation. The reposition of the parts prolapsed was accomplished in about ten minutes. Labor pains were rather slow for about three-quarters of an hour, and the patient, having been so very much fatigued by her uncomfortable posture, was permitted to lie on her left side with a high pillow under her hips. The pulsation of the child's heart, which had been very feeble, now recovered its full strength, labor pains re-appeared, the head engaged firmly in the pelvis, no further prolapsus occurred, and, at half-past twelve, a loud crying child (a boy, eleven pounds in weight) made its appearance. Childbed proceeded without the least disturbance. The child is now four months old.

Case III.—Mrs. Elizabeth Bohn, aged thirty-six, native of Germany, of vigorous frame, but somewhat reduced by a bronchial catarrh which persisted during the later months of gestation, sent for a midwife at eleven P. M., February 3d, 1860. The pains were so slow and feeble that patient and widwife slept several hours during the night. At five oclock A. M., contractions of the womb re-appeared more forcibly, the membranes ruptured, after which the midwife discovered the funis projecting through the os uteri, but could not find any fœtal part presenting. My assistance was called for at seven o'clock A. M., February 4th, 1860.

On examination, I found the funis in from four to five small loops projecting through the os uteri, which was only partially dilated; the head, being high above the pelvis, I could discover only by introducing my full hand.

After placing the patient in the position already described, efforts were made to replace the funis, which was more difficult in this case than in the two former, as several loops were projecting, and one would drop down while another was carried up; but nevertheless the aim was accomplished in a very short time, and the operating hand kept within the uterus in order to prevent another prolapsus. In the meantime, uterine contractions propelled the head into the pelvis. The patient was now placed once more on her back, (to her great comfort,) and ascultation soon convinced me of the child's life.

Labor proceeded rapidly, and at a quarter-past eight, A. M., one hour after my arrival, a crying child proclaimed to me once more the success of the operation.

The patient had some after-pains, which yielded to slight medication. She is now doing well.

A few remarks on the merits of the operation employed in the cases just reported, will be permitted.

The presentation, or rather the prolapsus of the funis, is by all authorities in the art of accouchment, considered as a complication most disastrous to the life of the fœtus, and the great variety of contrivances invented for the occurrence is the most eloquent testimony for the difficulty of its removal.

The space granted to this paper does not permit the reporter, nor is it his aim, to go into a detail of the various modes of treatment, but it may be remarked, that not only the life of the fœtus, but the life as well as health of the mother is often endangered or lost through the severe operation—forceps, version, and craniotomy—often resorted to, after repeated efforts have failed to replace the funis.

The following table, collected from the highest authorities, shows the numerical proportion of this occurrence:—

| | | | | |
|---|---|---|---|---|
| Collins, | 16152 cases of labor. | 97 funis presentations. | 1 out of | 165 |
| Churchill, | 90983 " " " | 322 " " | 1 " | 282 |
| Michælis, | 2400 " " " | 27 " " | 1 " | 88 |
| Boivin, | 20357 " " " | 38 " " | 1 " | 535 |
| LaChapelle, | 15652 " " " | 41 " " | 1 " | 411 |
| Hardy and McClintock | 6702 " " " | 37 " " | 1 " | 181 |
| Klein, | 5490 " " " | 55 " " | 1 " | 100 |
| Barstch, | 4425 " " " | 16 " " | 1 " | 276 |
| Arneth, | 6608 " " " | 33 " " | 1 " | 200 |
| Skanzoni, | 8415 " " " | 29 " " | 1 " | 290 |

Out of 177,184 accouchments, which is the total amount of the figures just referred to, prolapsus of the funis occurred 695 times, giving a proportion of 1:264, showing that this anomaly is one of the most frequent disturbances of labor.

Another table will show the relative mortality of children born under these circumstances:

| | Prolapsus of Funis. | Children Still-born. |
|---|---|---|
| Mauriceau. | 39 | 15 |
| De la Motte, | 14 | 5 |
| Clarke, | 66 | 49 |
| Collins, | 97 | 24 |
| Churchill, | 322 | 220 |
| Hardy and McClintock, | 37 | 25 |
| LaChapelle, | 41 | 8 |
| Michælis, | 27 | 20 |
| Boivin, | 38 | 18 |
| Arneth, | 33 | 11 |
| Skanzoni, | 29 | 13 |
| | 743 | 408 |

Thus, 743 cases of prolapsus funis gave 408 still-born children, a proportion of 1:1.82, which shows clearly enough that accouchers have not been very successful in treating this kind of labor.

The rationale of the posture method being obvious to every skillful practitioner, we shall, in conclusion, try to give the indications for it.

First: The operation is only admissible as long as circulation exists in the funis; even if the circulation is feeble, it may soon be restored after the impediment is removed.

Second: The os uteri must be sufficiently ditated or dilatable.

Third: The liquor amnii must be partly retained; otherwise, if it should all have escaped, and the uterus be firmly contracted over the child's body, every effort for the reduction of the prolapsed funis would be in vain.

